# Managing transitions from external assistance: cross-national learning about sustaining effective coverage

**DOI:** 10.1093/heapol/czad101

**Published:** 2024-01-23

**Authors:** Zubin Cyrus Shroff, Susan P Sparkes, Ligia Paina, Maria Skarphedinsdottir, George Gotsadze, Henry Zakumumpa, Kun Tang, Prasadini N Perera, MyMai Yuan, Kara Hanson

**Affiliations:** World Health Organization, Department of Health Financing and Economics, World Health Organization, Avenue Appia 20, Geneva 1211, Switzerland; World Health Organization, Alliance for Health Policy and Systems Research, Avenue Appia 20, Geneva 1211, Switzerland; Department of International Health, Johns Hopkins University School of Public Health, Baltimore 21205, USA; UHC 2030, World Health Organization, Avenue Appia 20, Geneva 1211, Switzerland; Curatio International Foundation, Georgia and Ilia State University, 0179 Kavsadze str. 3, Office 5, Tbilisi, Georgia; Department of Health Policy, Planning and Management, Makerere University, School of Public Health, 7062 University Rd, Kampala, Uganda; Vanke School of Public Health, Tsinghua University, 30 Shuangqing Rd, Haidian District, Beijing 100190, China; Institute for Health Policy, 72, Park Street, Colombo 2, Sri Lanka; University of Peradeniya, Galaha Rd 20400, Peradeniya, Sri Lanka; World Health Organization, Department of Health Financing and Economics, World Health Organization, Avenue Appia 20, Geneva 1211, Switzerland; Faculty of Public Health and Policy, London School of Hygiene and Tropical Medicine, Keppel St, London WC1E 7HT, UK

**Keywords:** Donor transition, transition, sustainability, external assistance, UHC

## Abstract

The often-prominent role of external assistance in health financing in low- and middle-income countries raises the question of how such resources can enable the sustained or even expanded coverage of key health services and initiatives even after donor funding is no longer available. In response to this question, this paper analyses the process and outcomes of donor transitions in health—where countries or regions within countries are no longer eligible to receive grants or concessional loans from external sources based on eligibility criteria or change in donor policy. The comparative analysis of multiple donor transitions in four countries—China, Georgia, Sri Lanka and Uganda—identifies 16 factors related to policy actors, policy process, the content of donor-funded initiatives and the broader political-economic context that were associated with sustained coverage of previously donor supported interventions. From a contextual standpoint, these factors relate to favourable economic and political environments for domestic systems to prioritize coverage for donor-supported interventions. Clear and transparent transition processes also enabled a smoother transition. How the donor-supported initiatives and services were organized within the context of the overall health system was found to be critically important, both before and during the transition process. This includes a targeted approach to integrate, strengthen and align key elements of the governance, financing, input management and service delivery arrangements with domestic systems. The findings of this analysis have important implications for how both donors and country policy makers can better structure external assistance that enables sustained coverage regardless of the source of funding. In particular, donors can better support sustained coverage through supporting long-term structural and institutional reform, clear co-financing policies, ensuring alignment with local salary scales and engaging with communities to ensure a continued focus on equitable access post-transition.

Key messages

- Donor transitions raise the critical question of how to sustain coverage of key health services in the absence of external assistance. It is essential to learn from experiences on how and why coverage is sustained post-donor transitions to better inform effective domestic and donor policies.
- Through the comparative analysis of nine donor-funded initiatives in four low- and middle-income countries, this paper identifies factors related to policy actors, policy process, the content of donor-funded initiatives and the broader political-economic context that were associated with sustained coverage of previously donor-supported services and initiatives.
- The convergence of a favourable economic and political context, clear and transparent transition processes and targeted approaches towards integration and coordination of key elements of governance, financing, input management and service delivery arrangements with domestic systems were associated with sustained coverage post-transition.
- Donors can better support coverage sustainability through supporting long-term structural and institutional reform, clear co-financing policies, ensuring alignment with local salary scales and engaging with communities to ensure a continued focus on equity post-transition.

## Introduction

Given the prominent role of external assistance in health financing in low- and middle-income countries (LMICs) (World Health Organization, 2022), the question of how it is used to expand and sustain the coverage of key health services is of critical importance to countries, and the donors that support them, seeking to move towards Universal Health Coverage (UHC). The COVID-19 pandemic highlighted that sustaining the coverage of priority health services requires political and financial support committed to strengthening health system foundations regardless of whether the source of financing is domestic or external (Sparkes *et al.*, 2021). Donor transitions, where countries are no longer eligible to receive grants or concessional loans from external sources—based on eligibility criteria or change in donor policy—have provided a particular focal point, highlighting the challenge of how to sustain coverage of previously donor-supported services and initiatives in the absence of external funding. While specific policies related to transition and overall terminology differ across institutions, over the past decade, development partners have paid significant attention to establishing pre-defined income or disease thresholds as triggers for transition (McDade *et al.*, 2020; The Global Fund, 2022; Gavi, 2023) to ‘self-reliance’ of domestic systems (USAID, 2020), or to a future ‘without aid’ (Government of Ghana, 2019). To engage effectively in these critical decisions and processes, stakeholders need to better understand the political, economic and health system factors that enable coverage to be sustained in the context of transitioning from external assistance to domestic financing (Shroff *et al.*, 2022).

Against this backdrop, a research programme was launched in 2020 to better understand whether, how and under what circumstances the coverage of priority health services previously funded by donors has been sustained. The impetus for this programme stems from growing consensus around the principle that donor transitions should ‘maintain or even increase coverage for priority health services, including those currently supported with external funds’ (UHC2030, 2018). This definition emphasizes that transition is more than simply replacing external with domestic funds, and involves reconfiguring financing, governance, inputs and service delivery within domestic health systems to enable the maintenance of service coverage gains.

As part of this research programme, research teams based in China, Georgia, Sri Lanka and Uganda^1^ analysed multiple donor transition experiences in each country and answered the questions: (1) how does the cessation of external financing affect the coverage of interventions previously supported by donor funding? (2) what political, financing and health system factors influence whether coverage of the intervention/service was sustained once donor funding was no longer available? These analyses which were in the form of detailed project reports were synthesized in-depth for the purposes of this paper. This includes reports on Uganda (Zakumumpa, 2022), Sri Lanka (Institute for Health Policy, 2023), Georgia (Gotsadze and Uchaneishvili, 2022) and China (Tang *et al.*, 2022).

The synthesis of experiences from nine donor-funded initiatives in these countries fills a gap in the existing literature by placing donor transitions within the scope of the broader domestic health systems and considers multiple transition processes within each country. While some research on domestic policy responses and system adaptations to donor transitions exists (Bossert, 1990; Kutzin *et al.*, 2018; Yamey *et al.*, 2019), the literature to date with few exceptions has not yet examined transition both within and across country contexts to generate cross-cutting evidence (Bennett *et al.*, 2011; 2015; Ozawa *et al.*, 2016; Gotsadze *et al.*, 2019; Rodríguez *et al.*, 2021).

In this paper, we address this gap in the literature by bringing together lessons on whether and how the actors are involved, the process of transition, context-related transition factors and the content of the initiatives either enabled or constrained sustained coverage. This paper is divided into the following sections: (i) a brief overview of the research framework used across all country studies and for this synthesis; (ii) the data sources and methods used with an overview of the initiatives included in the analysis; (iii) findings presented based on the categories corresponding to the research framework; (iv) discussion and limitations; and (v) conclusion.

## Research framework

**Figure 1.**
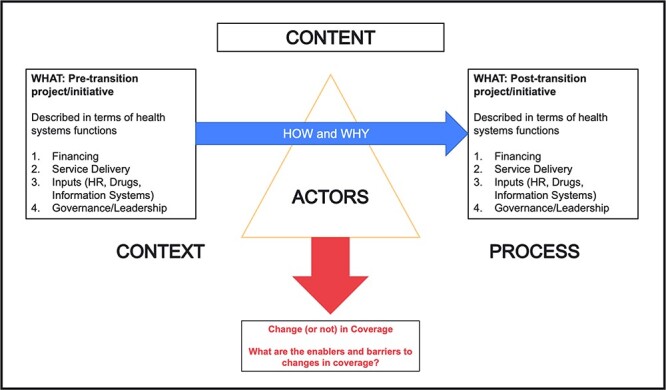
Research framework proposed in this paper

In line with the aims of this research programme, a framework based on an adaptation of the Walt and Gilson policy analysis triangle (Walt and Gilson, 1994) was developed by the authors (who also led the design of the research programme). The framework aimed to: (a) enable a common understanding of terms and concepts across the country studies, (b) allow for a systematic description of the donor-funded initiatives in terms of their organization around health system functions, (c) understand and analyse the context, process and actors surrounding the donor transition process, (d) elucidate the factors that influence and are linked with sustained coverage post transition and (e) facilitate cross-country analysis (World Health Organization, 2000). The schematic displayed in Figure 1 helps to illustrate the research programme’s overall perspective of the transition process.

The left-hand side box in Figure 1 describes the content of the donor-funded initiative prior to transition. Building from the health system’s functional framework and aligned with WHO’s work on cross-programmatic efficiency analysis (World Health Organization, 2000; Sparkes *et al.*, 2017), this includes content themes around financing (what resources were raised, how they were pooled and how providers were paid), service delivery (what services were delivered, by whom and how), inputs (how inputs including human resources for health, health information systems, and drugs and equipment were managed) and governance (what the governance and institutional arrangements were). The right-hand side box in Figure 1 describes these same elements in the post-transition period to understand whether there were changes to the actual content of these functions after transition. Building on concepts proposed by Walt and Gilson (1994) for policy analysis, we argue that the interplay of context (which includes the political, economic and social context), the content of the donor-funded initiative and its adaptation post transition, actors involved (including those within and beyond government) and the specific policy processes on transition play a major role in influencing whether and how coverage was sustained in the absence of donor funding.

The framework was operationalized through a series of guiding questions linked to each of the elements mentioned above and was used for both data collection and analysis. Recognizing the diversity of settings and initiatives included in the research programme, country teams selected to participate were encouraged to add questions as they saw relevant to the initiatives they were examining.

## Data sources and methods

At the time of writing, four country-level study reports, each analysing multiple donor transitions, had been prepared by the China, Georgia, Sri Lanka and Uganda research teams, using the common research framework described above^2^. Details of the initiatives included in each study, the disease condition they pertained to, initiative funders and period under examination are provided in Table 1. The four country studies covered nine donor-funded initiatives that are included in this analysis. The documented transitions, which took place between 1998 and 2018, were funded by a range of bilateral and multilateral donors including the Global Fund, Gavi, U.S. Agency for International Development (USAID), World Bank (WB), the U.S. President’s Emergency Plan for AIDS Relief (PEPFAR) and the UK Department for International Development (DFID; now the Foreign, Commonwealth and Development Office).

**Table 1.** Timeline of country initiatives

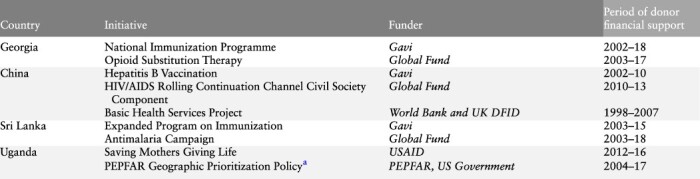

a The Geographic Prioritization Policy entailed PEPFAR exiting 10 ‘low- HIV-burden’ districts in Northern and Eastern Uganda in 2015 to focus its resources on geographic sub-regions with a higher HIV burden. The country study examined how this exit affected HIV-related services comparing districts impacted by this Geographic Prioritization policy with districts where PEPFAR continued to provide support. Informed by a deteriorating epidemiological picture PEPFAR in 2020 reversed this Geographic Prioritization policy.

Each of the four country reports included information on coverage objectives and whether they had been sustained post-transition. Reports also included data on the four elements of the framework: content (during both pre- and post-transition periods), context, actors and processes, drawing examples from across the initiatives to illustrate the role of these elements in influencing sustained coverage post-transition. The first two named authors independently went through the reports to develop initial codes related to each of the framework elements. Once a final list of codes was agreed upon the text of the country reports was systematically analysed to identify factors related to each of these elements that were reported to have influenced the sustainability of intervention coverage post-transition. These codes were then validated with the co-authors from each of the country teams to ensure accuracy. During this process we sought to identify the factors that enabled sustained coverage and those that constrained it post-transition by looking for both instances where factors were present and had positively influenced sustained coverage and instances where the absence of those same factors had negatively influenced coverage sustainability. Since the aim of the analysis was to identify common factors, only those factors that were present across more than one country were retained. The factors and examples illustrating them were then refined through iterative discussions among the author team with reference to the framework. The active engagement of authors from each of the country teams as co-authors ensured that the interpretation of the report findings was accurate and appropriate. As no data were collected for the purpose of the synthesis paper, no additional ethics review was sought beyond the approvals obtained by the country research teams from an ethical review board within that country. Details are available upon request from the authors.

## Findings

This section highlights some of the main findings that emerged through the cross-country analysis of the final reports submitted by country teams from China, Georgia, Sri Lanka and Uganda. The findings section begins with a summary of the coverage measures for each of the initiatives examined (Table 2). It then goes on to examine factors around transition related processes, the role of actors and context related factors that influenced sustained intervention coverage post-transition. Finally, factors associated with the content of the initiative are identified. Unless mentioned otherwise, all data referenced in the rest of this paper were taken directly from the country reports developed as part of the supported research programme. Country reports are available upon reasonable request to the country team authors.

**Table 2.** Initiative coverage, objectives and outcomes by country

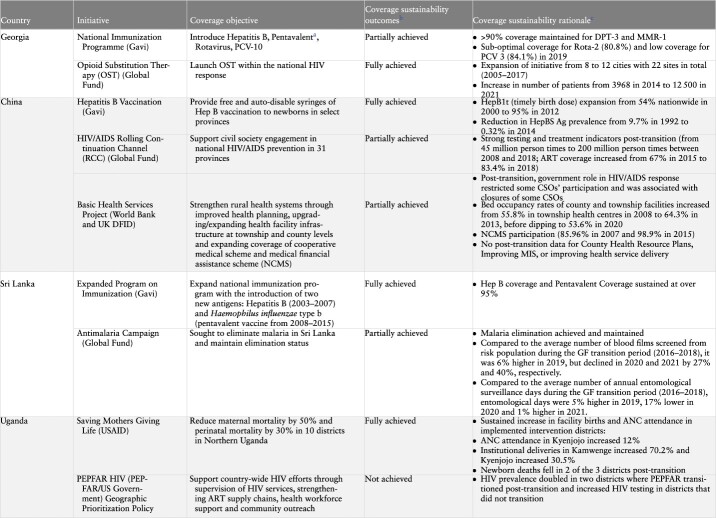

**Table 2.** (Continued)

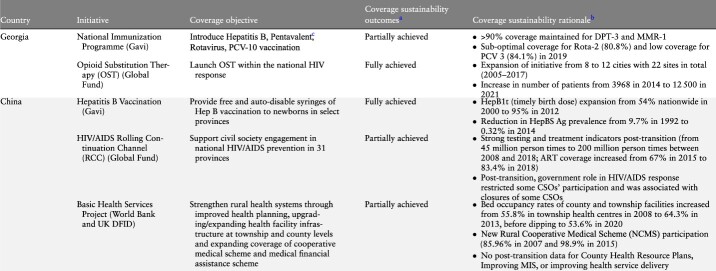

a Partially achieved refers to initiatives where some components and related coverage objectives were not fully achieved. Specific explanation for partial achievement is given in the column on ‘coverage sustainability rationale.’

b Coverage sustainability outcome indicators presented here were identified from the in-depth country case studies, as determined the authors of each case study, based on their specific relevance to the particular donor-supported initiative.

c Replaced in 2015 by Hexavalent procured with national funds.

*Source:* Georgia Immunization data: Ministry of Internally Displaced Persons from the Occupied Territories, Labour, Health and Social Affairs of Georgia (2020). Data on opioid substitution therapy programme: Javakhishvili *et al.* (2006); Beselia *et al.* (2018) (data on number of OST sites); Ministry of Internally Displaced Persons from the Occupied Territories, Labour, Health and Social Affairs of Georgia (2022) (data on number of patients on OST). China Hepatitis B data: Liu *et al.* (2019); Cui *et al.* (2017). HIV/AIDS testing and treatment indicators and ART coverage: National Health Commission of the PRC (2019). Data on bed occupancy rates at township and county facilities: National Health Commission of the PRC (2009; 2014; 2021). Data on NCMS participation: Gu *et al.* (2007). Sri Lanka Immunization data: WHO/UNICEF Estimates of National Immunization Coverage (WUENIC) (2023). Data on AMC: Anti-Malaria Campaign in the Ministry of Health of Sri Lanka. Uganda SMGL programme indicators: Uganda District Health Information System (DHIS-2) data. PEPFAR: HIV testing data: Uganda District Health Information System (DHIS-2) data. HIV prevalence data at district level: Ministry of Health Estimates, District HIV prevalence as of 31 December 2020.

### Summary of coverage measures for study initiatives

Table 2 provides an overview of the performance of individual initiatives in relation to the service coverage objectives that they sought to achieve. Of the nine initiatives, four were largely successful in achieving their coverage objectives: Gavi support to China and Sri Lanka in the areas of Hepatitis B and the expanded programme on immunization (EPI) respectively; Global Fund support to Georgia in the area of opioid substitution therapy (OST) and the USAID supported Saving Mothers Giving Life (SMGL) initiative in Uganda focused on reproductive, maternal, newborn, child and adolescent health (RMNCH). On the other hand, not all desired coverage outcomes were sustained with respect to several initiatives including Gavi support to the National Immunization Program (NIP) in Georgia, the Global Fund’s Rolling Continuation Channel (RCC’s) engagement of civil society groups in China, the Antimalaria Campaign in Sri Lanka and the WB/DFID supported Basic Health Services Project (BHS) initiative in China. PEPFAR in Uganda represents an initiative that did not achieve its coverage objectives.

### Explanatory factors for sustained coverage

Table 3 presents the list of transition-related factors that were identified to be associated with sustained coverage along with definitions for each of these. Table 4 presents the comprehensive list of factors and how these were present across each of the nine initiatives.

**Table 3.** Factors and their definitions

Framework category	Factor	Definition
Actors	Policy leaders and advocates within and outside government	Policy leaders and advocates (programme implementers, government officials, CSOs, patient groups, professional associations) played an important role in making the case with governments to replace lost donor funding
	Provision of technical support by donors	Donors played a role other than the provision of funding. In particular, donor’s roles evolved, and moved from a largely financial support role to one prioritizing technical contributions. This included post transition technical support and/or a role in bringing about ideational shifts in the country
Process	Transparency around transition processes	Transition processes were perceived by most respondents to have been carried out transparently and with open discussions
	Clear timelines and well-developed transition plans	Transition processes had clear and well-established timelines accompanied by detailed transition plans
Context	Economic growth	National level economic growth enabled the government to take over financial support to the programme once donor funding ceased
	Aligned with broader political agenda	The political agenda of either the Ministry of Health (MOH), Ministry of Finance (MOF) or other arm of government, was aligned with and supportive of both the services and populations receiving donor support
Content-financing	Clear approach and guidance around financing human resources (HR) and recurrent costs	The donor-funded project had a clear policy with respect to support for HR and other recurrent costs, whereby the domestic funds covered recurrent costs and donor funds paid for commodities/capital expenses
	On budget support which was in part enabled by strengthened public financial management systems (PFMs)	On budget donor-funded support and support for strengthening of domestic PFM systems
	Clear approach to co-financing	The donor-funded project was marked by a clearly elucidated co-financing policy from inception or early in the project
Content-governance	Early integration/streamlining of project governance with regular government arrangements	Project governance was integrated/streamlined with the existing government apparatus and arrangements from project inception/early on in the project
	Participatory and inclusive governance arrangements	The governance structures established during the donor-funded project/ transition process were participatory involving stakeholders from different parts of the MOH/government/beyond government
	Inclusion of legal and regulatory reform support	Legal/regulatory reform support (for things such as PFM, supply chains, HRH, procurement) was provided as part of donor support during the period of the project/transition
Content- inputs and service delivery	Avoiding establishing parallel systems	The donor-funded project did not establish parallel systems for the management of inputs (human resources for health (HRH), procurement of drug and supplies, information systems), and when such systems were established they were gradually integrated into the national systems
	Purposeful use of resources for institutional strengthening in the long run	Resources during the course of the donor-funded project/transition were used to strengthen institutions and processes around input management (including for monitoring and evaluation, PFM reform, supply chain management, improving HRH recruitment and management practices, strengthening information systems)
	Consolidating procurement	The donor-funded project/transition period was used as an opportunity to consolidate procurement across different programmes/ integrate procurement within a single agency/structure
	Use of established service delivery channels	Services funded through the donor-funded project were provided through already established service delivery channels (in other words, service delivery was not carried out separate from pre-existing channels and systems)

**Table 4.** Factors associated with sustained coverage^a^

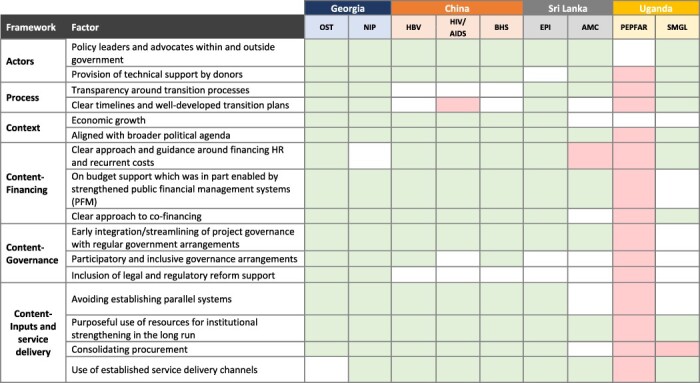

a Green: the presence of this factor was reported to be positively associated with sustained coverage in the given initiative; red: the absence of this factor was reported to be negatively associated with sustained coverage in the given initiative; white: the factor was not identified as either present and positively associated with sustained coverage or absent and negatively associated with sustained coverage.

## Actors

Through their engagement in policy processes, actors play a major role in bringing about changes in the content of initiatives. The country studies illustrate the importance of two actor groups in sustained coverage over time, namely (a) policy leaders and advocates within and outside government and (b) donors and technical partners.

### Policy leaders and advocates within and outside government can play a major role in sustaining coverage post-transition

In the case of the SMGL initiative in Uganda, sub-national implementers were identified as important policy champions who made the case for continued support to the initiative post-transition. The Sri Lanka study noted the support from officials at the Ministry of Finance in ensuring stable financial support for the EPI, a key enabler of sustained coverage. Outside of government, Civil Society Organizations (CSOs) can play an important role in raising awareness and mobilizing communities to put pressure on governments to ramp up funding for and thus sustain intervention coverage.

### Donors and external technical/implementing partners, through the provision of technical support, can contribute to sustaining coverage

In particular, the country studies point to how their role evolves, moving from a largely financial support role to one prioritizing their technical contributions. The China study highlights the influential role of donors, particularly the WB which through its role as what respondents termed ‘a developmental knowledge bank’ with a history of support to China was able to influence Chinese policy and practice long after financial project support ended. In the case of the Global Fund’s support to China’s CSOs under the RCC initiative, the study reports that the project was successful in bringing about ‘ideational convergence between China and the global HIV/AIDS paradigm’ emphasizing de-stigmatization, inclusion and community involvement in a way not seen earlier. The importance of continued technical support following the end of financial support was also highlighted in the case of the SMGL initiative in Uganda where the technical engagement by USAID-appointed implementing agencies post transition was underscored as an important enabler of sustained coverage.

## Process

In addition to influencing the content of policies, decision-making processes have a major role in influencing their implementation including at the level of communities. Two process factors identified in the analysis were: (a) transition discussions characterized by transparency and trust and (b) clear timelines around transition with well-developed transition plans.

### Transparency around transition processes positively influenced transition outcomes

The importance of transparency and trust is highlighted by the Uganda study in which respondents noted disagreement between PEPFAR and government officials on the data that were used to justify transitioning out of ‘low intensity’ HIV districts, with negative long-term implications for HIV control. In contrast, open discussions through long standing immunization summits convening officials from the MOH, MOF and physician groups around the EPI in Sri Lanka were reported to have played a major role in building MOF trust in and support for EPI in the country.

### Clear timelines and well-developed transition plans enabled successful transitions

Well-developed transition plans with clearly defined roles and responsibilities and well-defined timelines were observed to have enabled smooth transitions across initiatives studied in Georgia, the EPI initiative in Sri Lanka and the SMGL initiative in Uganda. By contrast, the sudden loss of funding in China’s Global Fund-supported RCC initiative (on account of the country being deemed ineligible to receive funding due to its economic performance) was reported to have negatively impacted the ability of CSOs to reach out to marginalized populations with implications for community empowerment.

## Context

The context within which a donor transition takes place is endogenous to the other categories in the framework and to the outcomes of the transition process more broadly. These contextual factors relate to both the economic and political climate within which the donor transition takes place. The country studies highlight the role of (a) economic growth and (b) alignment of initiatives with broader political agendas.

### Rapid economic growth was often associated with successful transitions

Economic growth in several of the countries was found to be critical in terms of releasing fiscal pressures to fund previously donor-supported services. This is specifically relevant for China, Georgia and the EPI transition in Sri Lanka, where transition was triggered in all cases by economic growth in accordance with the policies of the concerned funder (Heller, 2006; The Global Fund, 2022; Gavi, 2023; World Bank, 2023). A counterpoint to this experience is in Uganda, where consistent but relatively slow economic growth has meant that external assistance continues to play a large role in health financing in the country and where consequently transition is more elusive. External assistance accounted for 43% of current health expenditure in Uganda in 2019, compared to 1% in Georgia, 2% in Sri Lanka and <1% in China (World Health Organization, 2023). Despite its importance, it is not the sole significant factor in explaining successful transition, particularly given the within-country variation in coverage sustainability as demonstrated in all four countries included in this study. In each of the countries, there were certain activities or coverage elements that were not sustained following transition despite strong economic growth.

### Coverage sustainability was highly influenced by alignment with broader political agendas and more specifically with whether or not the political agenda of the Ministry of Health (MOH), Ministry of Finance (MOF) or executive was aligned with and supportive of both the services and populations receiving donor support

Importantly, this political support was not necessarily present at the start of the donor support, which in many of the initiatives prioritized marginalized populations. Rather, it had to, in these instances, be built over the course of the initiative, including through community engagement. This factor was found to be particularly important in the transition process for OST in Georgia, Global Fund support for CSOs as part of the RCC in China, and the USAID-supported SMGL initiative in Uganda. In contrast, it was found in Uganda that at the community level people were not empowered to demand government action to continue support for PEPFAR-related HIV services and therefore coverage was not sustained in some areas.

In some of the initiatives, higher-level political support was important both pre- and during transition. For example, Sri Lanka’s commitment to eliminating malaria represented a government-wide commitment. China’s expansion of Hepatitis B vaccination was noted to have been spurred by a WHO report focusing on the country’s vaccine financing landscape leading to high-level political commitment to addressing it. In the case of Georgia, several of the positive changes around input management, health financing and service delivery were catalysed by an eagerness to align with European Union (EU) standards in line with the country’s EU membership aspirations.

## Content

The comparative analysis highlighted several areas where the content of donor-funded initiatives either enabled or constrained service coverage in the post-transition period. Our analysis examines factors around three areas related to content: (a) financing, (b) governance and (c) inputs and service delivery. While the findings are presented based on function, there is inevitable convergence as to how each initiative was functionally organized. As a result, overlapping themes emerge in this in relation to the initiative content.

### Financing

The country studies highlighted the mechanics of financing arrangements as critical to sustaining service coverage and enabling integration of donor-supported initiatives into domestically financed systems We identify three financing-related factors that are associated with successful transition experiences: (a) a differentiated approach and separation of salaries and other recurrent costs between domestic and external financing, (b) the strengthening of public financial management systems (PFM) and on-budget allocations and (c) a clear approach to co-financing.

### Clear guidance and consensus on how donor financing was to be used before and during the transition, as well as not using these funds to support recurrent costs, impacted how smoothly domestic, public financing was able to replace aspects of donor financing

For example, from the outset of Gavi support in Sri Lanka, the government used external funds only to pay for the vaccines themselves, and all human resources and service delivery-related costs were financed with domestic public resources. Additionally, paying health worker salaries primarily out of domestic resources and ensuring alignment of salaries and other recurrent costs with local salary scales were key enablers to the transition process in Georgia’s transition from Global Fund support for OST and Uganda’s transition of the SMGL initiative. On the other hand, as demonstrated by the PEPFAR initiative in Uganda, the creation of new cadres of health workers that were not represented in the public system was associated with significant health-worker attrition post-transition.

### Placing donor funds on-budget both in advance of and during the transition process made it easier for domestic, public funds to replace donor funding post-transition

Initiatives such as the WB/DFID-supported BHS Project in China placed external assistance on-budget from the outset ensuring that public financial management capacity was in place to connect funds with requisite services. In the case of the Global Fund transition in Georgia, the government worked with both the Global Fund and other partners to strengthen the underlying public financial management arrangements in the health sector to ensure that domestic funds could support relevant services, including OST. This included processes around the medium-term budgeting process and the introduction of programme-based budgeting. In contrast, resources for Uganda’s PEPFAR initiative were off budget. In this case, insufficient attention to domestic budget and political prioritization threatened service coverage post-transition.

### A clear policy on co-financing^3^ (whether from the country or donor) emerged as an enabler for maintaining service coverage during transition

In connection with the process of transition, both the Georgia and Sri Lanka studies referenced Gavi’s clear policy related to the co-financing of vaccine costs as an enabler of sustained coverage post transition. In Georgia, while the Global Fund also had a policy related to co-financing, it was less clear cut in terms of amounts and modalities. Therefore, while it facilitated domestic financial buy-in to the OST initiative, the specific costs to be paid for with domestic, public funds were less well-defined.

### Governance

Factors around the governance of initiatives at national, subnational and facility levels that were associated with successful transitions included (a) early integration of project governance arrangements within the routine government apparatus, (b) the establishment of participatory governance arrangements engaging diverse stakeholders and (c) support for legal and regulatory reform.

#### Early integration of project governance with existing governance arrangements was associated with sustained coverage

The China and Georgia cases demonstrate the important role of the early integration of project governance arrangements into the routine government apparatus in successful transitions. The ‘one office, two function approach’ in China that integrated project functions within existing government offices at the national and sub-national levels illustrated this. Project-designated staff received the same salary as regular staff and continued the same function post transition as they did during the period of donor support. In Sri Lanka, Gavi’s additional support to the EPI was managed under the established EPI structure with no separate apparatus put in place, which was also the case for Gavi’s support to Georgia’s NIP.

#### Participatory and inclusive governance arrangements positively influenced sustained service coverage post-transition

The studies demonstrate the importance of participatory and inclusive governance arrangements resulting in increased acceptance of decisions taken and contributing to long-term sustainability. In Georgia, the country study noted the importance of inclusive national dialogue and participatory decision-making by a broad group of stakeholders through coordinating bodies and decision-making mechanisms including Inter-Sectoral Collaboration Committees, the Country Cooperation Mechanism (CCM) and the Policy and Advocacy Advisory Council. Decisions taken by these bodies were able to reflect the opinions of diverse stakeholders, something that in turn fed through to national policy and practice. However, governance arrangements that were not aligned with local political systems were typically not sustained post-transition. Illustrative of this, in China, the Global Fund’s institutionalized engagement of organizations of people living with HIV/AIDS and of HIV/AIDS related grassroots CSOs though their participation in the CCM was discontinued post-transition. While this discontinuation had a negative effect on the empowerment of community-based CSOs, the precedent of involving CSOs through the CCM set the foundation for select CSOs to be invited by the government to serve on the Advisory Committee of the China Aids Fund for NGOs.

#### Legal and regulatory reforms are crucial enablers of successful transitions

Successful transitions are also enabled by legal and regulatory reforms that pave the way for the incorporation of project-supported activities into routine government programmes and policies. In the case of Georgia’s OST initiative, the passage of the 2002 Law on Narcotic Drugs, Psychotropic Substances and Precursors and Narcological Assistance was mentioned as a major milestone. A working group established as part of an EU-funded project on harmonization of national legislative and regulatory frameworks played a significant role in the development of the law. In the case of Georgia’s NIP, legal and regulatory changes were observed to have played a major role in easing the purchase of high-quality vaccines. This included legislation allowing vaccines to be procured through UNICEF procurement mechanisms as an alternative to state procurement, thus enabling these products to be made available in Georgia without further registration (Gavi, 2015).

### Inputs and service delivery

Four factors around input management and service delivery were associated with sustained coverage post-transition. These include: (a) avoiding the establishment of parallel systems around information, supply chains or the health workforce, (b) the strategic use of resources for institutional strengthening, (c) consolidating procurement and (d) delivering services through established channels and facilities, whereby multiple services were provided.

#### Projects that were successfully transitioned tended to avoid establishing parallel systems whether for HRH, information systems or procurement

In Georgia’s NIP initiative, reporting, management and procurement were all integrated into the national system and Gavi did not provide salary top-ups to health workers. Gavi also used existing cold chain systems and did not develop any new parallel cold chain infrastructure exclusively for its initiative. In Sri Lanka, Gavi did not provide any parallel funding for human resources and the initiative did not support any personnel dedicated exclusively for vaccination. In addition, the initiative used the existing EPI information systems while deploying new vaccines, averting the need for any purposeful integration of this function later on. This was also the case for all the initiatives examined in China.

#### Successful transition experiences were also characterized by a purposeful use of resources to strengthen health system capacities

All three initiatives in China and the SMGL initiative in Uganda played a major role in strengthening monitoring and evaluation systems, and practices acquired during the project period were retained post-transition. The Georgia study highlights how donor support (in this case from another donor, the EU) was deployed to strengthen the health management information system; this enabled availability of high-quality real-time data at different levels of the system, from which the initiatives (particularly OST) were able to benefit.

#### Initiatives where procurement of drugs and supplies was consolidated were associated with sustained coverage

In Sri Lanka, the study noted that centralized procurement enabled equitable access to vaccines by ensuring they were available where needed most. It was also noted to potentially enhance financial sustainability by providing Sri Lanka with the market power needed to obtain lower vaccine prices than even those negotiated by Gavi. In Georgia, the consolidation of vaccine procurement within the National Center for Disease Control and Public Health (NCDC) coupled with the strengthening of NCDC’s capacity for vaccine planning, budgeting and procurement was reported to have been associated with enhanced procurement processes post transition. In both Sri Lanka and Georgia, the push to consolidate procurement was driven by governments.

#### Delivering services through established channels and facilities, whereby multiple services were provided, enabled a smoother coverage transition

In the case of Gavi-supported initiatives in Georgia, Sri Lanka and China, the newly introduced vaccines were delivered through vaccination programmes that were well-established, as well as integrated with overall maternal and child health programmes. Given the strength of Sri Lanka’s EPI and maternal and child health system, the MOH ensured that no structural changes would be required to deliver Gavi-supported vaccines. The SMGL initiative in Uganda also took an integrated approach from its inception, by enabling infrastructure upgrades to existing maternal and neonatal care facilities. While this approach worked to leverage existing infrastructure, it also proved to be a challenge to fully maintain the cost and upkeep of new equipment and facilities post-transition. A similar constraint was faced in the PEPFAR transition in Uganda, where even though community outreach teams were embedded within existing community health systems through Village Health Teams, the absence of funding for allowances meant that it was difficult to maintain the priority for these activities post-transition.

## Discussion

The experiences of these nine initiatives across the four countries suggest that the convergence of a favourable economic and political context together with elements of governance, financing, input management and service delivery arrangements and transition processes were associated with sustained coverage post-transition. While no single factor was found to be *deterministic* of sustained coverage, we find that initiatives where a higher number of factors were present tended to do better than those where fewer factors were present (see Table 4). In general, we find that comparative analysis, both within countries and across countries, provides varied experiences that when applied to the research framework enable the identification of factors that either enable or constrain sustained coverage. By controlling for context-specific factors such as level of development, examining the variation in coverage across donor-funded initiatives *within* countries post-transition provides a particularly strong basis to identify and compare the role and intensity of these factors in determining transition outcomes. There is clear interaction and convergence across the specific categories, whereby context, actors, process and content are all interrelated in terms of ultimately sustaining coverage. In this way, the findings above cannot be taken in isolation from one another for any single initiative or country. In this discussion, we pull out themes that cut across these categories and can be considered by both donors and countries in terms of factors enabling sustainable coverage.

The contrasting experience of Georgia’s OST initiative when compared to the experience of PEPFAR in Uganda is illustrative of these findings. The OST initiative’s transition occurred against a backdrop of rapid economic growth and Georgia’s geopolitical aspirations to align itself with the EU, along with a vocal civil society community advocating for these services. Transition processes were characterized by transparency and high priority was given to (a) integration of the initiative’s financial, supply chain and human resources with the underlying domestic health system, as well as (b) strengthening of and technical assistance for these underlying domestic health system functions. By contrast, the implementation of PEPFAR in Uganda remained truly ‘verticalized’ in terms of its organization and interaction with the underlying domestic health system, which was found to have negative consequences for sustained coverage post-transition. The Ugandan government’s reluctance to step in and provide funds to maintain services post-transition (in contrast to the SMGL initiative focused on RMNCH services) also demonstrate the challenge of sustaining coverage gains where initiatives are not coordinated or integrated with the overall health system and where the transition process is not clear or transparent. Finally, transition processes for PEPFAR’s Geographic Prioritization policy were characterized by contestation over the data used to inform the transition policy and process, exacerbating the unequal power relationship between donor and recipient in a highly aid dependent context.

Our analysis demonstrates the importance of the alignment of domestic and external priorities, not just in terms of the disease condition supported through the initiative but also in terms of the approach adopted. The Global Fund’s vision of empowered CSOs playing a role in decision-making around HIV policy in China was fundamentally at odds with the government’s view of them as service providers with little agency of their own. Thus, while HIV testing and treatment rates were sustained and even increased post-transition, a number of CSOs stopped functioning after the Global Fund’s exit and the country’s HIV programme faced challenges in accessing the most vulnerable and hard-to-reach groups.

Donor supported initiatives are thus more likely to successfully transition if they are aligned to a country’s socio-political context. This finding resonates with recent work on donor transition in middle-income countries, which also stresses the importance of political will in determining coverage outcomes (Huffstetler *et al.*, 2022). However, it is important to note that context is itself not static and external assistance and its related initiatives can, in some instances, be catalytic of long-term ideational change. For example, the support for domestic funding for Georgia’s OST initiative was spurred by the combination of donor-supported legal reform and community mobilization towards decriminalization of injecting drug use. Similarly, the WB’s emphasis on training and high-quality evidence to inform the work of the BHS initiative was said to have been critical for creating a culture of learning and use of research to inform policy design and implementation in China.

In terms of transition processes, we note the relative absence of discussions around the equitable nature of coverage post-transition. This is particularly important as several donor initiatives specifically target hard-to-reach and other vulnerable and marginalized populations. Inclusive transition processes that seek to ensure that equity of coverage is sustained post transition are thus necessary. Engaging CSOs on an ongoing basis and ensuring genuine community participation are two possible ways of enabling this.

This analysis has several implications for the design of donor-funded initiatives and for the arrangements that will eventually transition them. First, there is an often-repeated need for donors to avoid competing and parallel arrangements for their initiatives, for example around financing, supply chains or human resources. However, donors need to go beyond just ‘doing no harm’ to actively support strengthening systems. This entails engaging and positioning initiatives within the overall foundational functions of the health system within which donor-supported initiatives are implemented, as well as allocating funding for long-term structural reforms, for example around public financial management systems or legal and regulatory reforms. This longer-term perspective has implications for the modality through which donor funds are disbursed. These studies also show that a more explicit approach is needed to the type of costs that can be supported with donor funds. For instance, rather than funding entire initiatives, there is scope for supporting specific elements (such as capital investments) that may enable donor funding to better complement (rather than substitute) domestic financing. And where recurrent/operational costs are supported with external funding, there are clear indications that actively aligning these inputs to ‘business as usual’, such as using local grades and salary scales, is important to enable aligned and complementary investments that can be clearly supported with domestic, public funds after transition. Finally, in many settings, donors have a major role in working with CSOs and patient groups towards building coalitions for change. In addition to mobilizing communities to make the case for increased investments in health, such coalitions also have a role in using political windows of opportunity to bring about policy change including through the strategic use of evidence and enabling integrated and aligned approaches to donor assistance that facilitate sustained coverage. Peer exchange and knowledge sharing on good practice are also enablers of sustained coverage. Further analysis is also called for to better understand how and for which priorities external funding may best complement public domestic resources for health.

The availability of funding and existing capacities means that many of these practices are undoubtedly easier to implement in middle income countries than those lower down the income ladder. However, as the experience of Uganda demonstrates, income levels do not necessarily play a deterministic role. The political prioritization of RMNCH, including through advocacy by sub-national level actors, was associated with increased government support for the SMGL initiative. This was in sharp contrast to the absence of government support for HIV/AIDS services once PEPFAR withdrew funding to specific districts in line with its Geographic Prioritization policy. Within-country analyses from China, Georgia and Sri Lanka also clearly demonstrate the importance of factors beyond GDP in influencing sustained coverage.

Finally, this analysis also draws attention to the importance of a country’s historical and geopolitical context in informing actor behaviour and policy processes that in turn influence coverage sustainability. Sri Lanka’s history of political priority for health had resulted in earlier experiences of fully transitioning the EPI to domestic funding as far back as the 1990s. This was identified as a significant factor in facilitating the later Gavi transition for more recently introduced vaccines that are examined in this research programme. Similarly, Georgia’s geopolitical ambitions for EU membership enabled significant financial and technical assistance that facilitated transition. By contrast, Uganda’s often difficult history with donors and lack of priority for health while continuing to be dependent on external resources has often reinforced fragmentation of resources. Correspondingly, donors continue to provide support through vertical initiatives accompanied by hierarchical policy processes that reinforce unequal relationships between donors and national-level stakeholders (Oliveira Cruz and McPake, 2011; Nannini *et al.*, 2022).

There are limitations to this study. From a comparative perspective, the initiatives analysed had primarily favourable outcomes in terms of sustained coverage. This could be a matter of selection of the initiatives chosen to be covered, as well as those that were eligible for transition. Second, the precision of estimating pre- and post-transition time horizons can differ by country and initiative based on interpretation by the study teams. There is particularly an issue of defining what constitutes a post-transition period given the continuation of technical assistance in most settings even when direct financial support for initiatives had ceased. Third, the focus of this paper on identifying factors related to sustained coverage across country contexts limits our ability to carry out in-depth analyses of specific political and economic incentives that shape actor behaviour and policy processes as mentioned in the paragraph above. We hope that future research can continue to document transitions and better understand how and why outcomes are sustained in the post-transition period. We also hope that future research will do more to advance theoretical frameworks that serve not just to identify factors associated with sustained coverage (as the framework outlined in this paper has sought to do), but also to test specific hypotheses around how actors, domestic and international political economy interact in particular contexts to produce the coverage outcomes we see, something that will greatly enrich our understanding of transitions and aid sustainability more broadly.

## Conclusion

The need to design external assistance to best enable sustained coverage has been a topic of discussion for many years. By systematically comparing four country experiences in managing multiple donor transitions from the health system perspective, this study provides concrete indications that are relevant specifically for donor transition, but also for the design of external assistance more broadly. While the context within which these transitions occurred was influential, it was not deterministic in isolation from factors more specifically related to how the initiatives were structured and the overall strength of the health system, in terms of contributing to sustained coverage. This finding shows the importance of deliberate and careful design of external assistance programmes and the importance of domestic political engagement for the achievements of externally supported initiatives to be sustained.

## Footnotes

1. Country teams were selected for this research programme based on an open call for proposals issued in December 2020. Teams were selected based on the strength of their research proposals as assessed through an external peer review process while ensuring diversity in terms of geography and country income group (upper-middle-income, lower-middle-income countries and low-income countries).

2. Five of the ten authors of this synthesis paper supported the conceptualization and development of the overall research programme under which the four country reports were developed. This includes development of the protocols for each of the country studies, provision of detailed feedback on interview guides developed by the country teams and extensive review of intermediate and final reports developed by the country teams over a two-year period. They are therefore intimately acquainted and have worked extensively with each of the four country studies.

3. Co-financing refers to the amount of a donor-funded project a country agrees to fund from domestic, public sources in relation to donor funds.

## Abbreviations

AMC = Antimalaria Campaign

CCM = Country Cooperation Mechanism

CSO = Civil society organizations

DFID = UK Department for International Development

EPI = Expanded Programme on Immunization

EU = European Union

MOF = Ministry of Finance

MOH = Ministry of Health

NCDC = National Center for Disease Control and Public Health

NIP = National Immunization Program

OST = Opioid Substitution Therapy

PEPFAR = U.S. President’s Emergency Plan for AIDS Relief

PFM = Public Financial Management

RCC = Rolling Continuation Channel

RMNCH = Reproductive, Maternal, Newborn, Child and Adolescent health

SMGL = Saving Mothers Giving Life

UHC = Universal health coverage

USAID = United States Agency for International Development

WB = World Bank

## Reflexivity statement

This paper brings together findings from a multi-country research programme that in addition to developing high quality health systems research related to donor transitions within the context of domestic systems sought to encourage research ownership by LMIC researchers. The list of authors includes researchers from each of the country study teams analysed in the paper. Six of the 10 authors are nationals of LMICs, all of whom have experience living and working in LMICs. Six of the ten authors are women. The author team includes individuals with diverse levels of experience and seniority. The diversity of the authors has enabled this paper to bring together a range of viewpoints and reflections including those based on lived experience.
